# Serum Metabolomics Based on GC-MS Reveals the Antipyretic Mechanism of Ellagic Acid in a Rat Model

**DOI:** 10.3390/metabo12060479

**Published:** 2022-05-25

**Authors:** Fengfeng Xie, Liba Xu, Hua Zhu, Yinlan Li, Lizhen Nong, Yaling Chen, Yanfang Zeng, Sijie Cen

**Affiliations:** 1 School of Chemistry and Chemical Engineering, Guangxi MinZu University, Nanning 530006, China; 15177143553@163.com or; 2 Guangxi Engineering Research Center of Ethnic Medicine Resources and Application, Guangxi Key Laboratory of Zhuang and Yao Ethnic Medicine, Collaborative Innovation Center of Zhuang and Yao Ethnic Medicine, Guangxi University of Chinese Medicine, Nanning 530200, China; xuliba15078772841@163.com (L.X.); yinlan.li@beigene.com (Y.L.); nonglizhen10086@163.com (L.N.); xingyun170210@163.com (Y.C.); yanfang.zeng@sanofi.com (Y.Z.); censijie0211@163.com (S.C.)

**Keywords:** fever, metabolomics, gas chromatograph-mass spectrometer, ellagic acid, Western blot, IKB-α

## Abstract

Ellagic acid (EA) is a polyphenol dilactone that has been reported to have antipyretic, anti-inflammatory, anti-tumor, and antioxidant activities, but the mechanism of action has not been reported. In this study, serum metabolomics was used to explore the mechanism of EA on rat fever induced by beer yeast, and to screen out marker metabolites to provide a reference for the antipyretic effect of EA. The acute fever model of male Sprague Dawley rats involved subcutaneous injection with 20% aqueous suspension of yeast (15 mL/kg) in their back. At the same time of modeling, EA was given orally by 10 mL/kg intragastric administration for treatment. During the experiment, the temperature and its change values of rats were recorded, and Interleukin-6 (IL-6), Tumor Necrosis Factor-α (TNF-α), Prostaglandin E2 (PGE2), Cyclic Adenosine Monophosphate (cAMP), Superoxide Dismutase (SOD) and Malondialdehyde (MDA)—six physiological and biochemical indexes of rats—were detected after the experiment. In addition, the hypothalamus of each rat was analyzed by Western blot (WB), and the levels of Phospho Nuclear Factor kappa-B (P-NF-κB P65) and IkappaB-alpha (IKB-α) were detected. Then, the serum metabolites of rats in each group were detected and analyzed by gas chromatograph mass spectrometry and the multivariate statistical analysis method. Finally, when screening for differential metabolites, the potential target metabolic pathway of drug intervention was screened for through the enrichment analysis of differential metabolites. Pearson correlation analysis was used to systematically characterize the relationship between biomarkers and pharmacodynamic indicators. EA could reduce the temperature and its change value in yeast induced fever rats after 18 h (*p* < 0.05). The level of IL-6, TNF-α, PGE2, cAMP, SOD and MDA of the Model group (MG) increased significantly compared to the Normal group (NG) (*p* < 0.001) after EA treatment, while the levels of the six indexes in the serum and cerebrospinal fluid of yeast-induced rats decreased. The administration of yeast led to a significant increase in Hypothalamus P-NF-κB P65 and IKB-α levels. Treatment with EA led to a significant decrease in P-NF-κB P65 levels. Moreover, combined with VIP > 1 and *p* < 0.05 as screening criteria, the corresponding retention time and characteristic mass to charge ratio were compared with the NIST library, Match score > 80%, and a total of 15 differential metabolites were screened. EA administration significantly regulated 9 of 15 metabolites in rat serum. The 15 differential metabolites involved linoleic acid metabolism, phenylalanine, tyrosine and tryptophan biosynthesis, galactose metabolism, biosynthesis of unsaturated fatty acids and glycerolipid metabolism. Pharmacodynamic correlation analysis was conducted between 15 different metabolites and six detection indexes. There was a significant correlation between 13 metabolites and six detection indexes. D-(−)-lactic acid, glycerin, phosphoric acid, 5-oxo-L-proline were negatively correlated with TNF-α, and *p* values were statistically significant except for L-tyrosine. In addition, glycerin was negatively correlated with IL-6, PGE2 and MDA, while phosphoric acid was negatively correlated with IL-6. In conclusion, EA may play an antipyretic anti-inflammatory role through the inhibition of the IKB-α/NF-κB signaling pathway and five metabolic pathways, which may contribute to a further understanding of the therapeutic mechanisms of the fever of EA.

## 1. Introduction

Fever refers to the situation in which the pyrogen directly acts on the thermoregulatory center, the functional disorder of the body temperature center, or causes excessive heat production and reduced heat dissipation due to various reasons, resulting in the rise of body temperature beyond the normal range. It is the most commonly observed symptom caused by infection or inflammation in many diseases [1]. Fever is a common brain-mediated antitraumatic, infective, or non-infective inflammatory response mediated by endogenous pyrogens such as IL-6, TNF-α, PGE2, corticotropin-releasing factor, and endothelin-1 [2,3]. Experimental studies showed that PGE2 and cAMP were important central heat mediators [4,5]. NF-κB has been widely reported to play an important role in inflammatory response and catabolism [6,7]. The NF-κB pathway can be activated by a variety of stimuli, such as IL-1β, TNF-α, or by IL-1R, TNF-FR and TLRs in yeast. This ultimately stimulates the expression of MMP-1, IL-6, and IL-8, which enter the nucleus through phosphorylation of P65 and subsequent translation of P65 [8,9]. 

**Figure 1:**
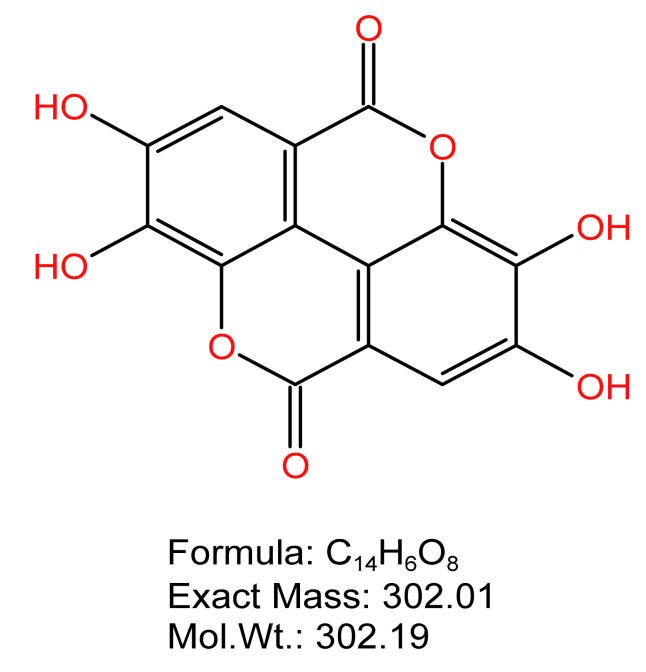
The structural formula of ellagic acid.

Ellagic acid (EA) is widely found in fruits and nuts. It is a natural plant polyphenol, usually in the form of yellow needle-like crystals, with a relative molecular weight of 302. 19 dalton (Da), molecular formula:C_14_H_6_O_8_ and high thermal stability. The structural formula of ellagic acid is shown in Figure 1. EA occurs naturally in condensed forms (such as ellagic tannins and glycosides), but also in free forms in the fruits of many plants [10]. A yeast heat-induced model can significantly increase the content of inflammatory factors in the serum of rats, and EA can reduce the concentration of TNF-α, IL-6 and IL-1β in serum, indicating that EA could inhibit the secretion of inflammatory cytokines and thus inhibit the occurrence of inflammation [11]. EA inhibited microglial pro-inflammatory activation and stimulated the release of the anti-inflammatory cytokine IL-10 under pro-inflammatory conditions and repress the activation of canonical inflammatory pathways, such as MAPK, NFAT and NF-kB, thus suggesting potential benefits for other inflammatory-associated dysfunctions [12]. In the dextran sodium sulfate (DSS)-induced model of chronic colitis, EA had the ability to prevent IKB-α degradation, inhibiting NF-κB P65 and STAT3 expression, thus acting as an anti-inflammatory [13]. In addition, it was found that EA has an antioxidant effect; it can protect the fetal kidneys of rats exposed to nicotine by decreasing MDA level and increasing SOD and GSH-Px activities [14]. 

Metabolites are the basis of organism phenotype and can help us to understand biological processes and mechanisms more intuitively and effectively. Based on qualitative and quantitative analysis of metabolites, metabolomics can be used to analyze metabolic pathways or metabolic networks, study the metabolic basis of macroscopic phenotypic phenomena of different organisms [15,16], response mechanisms of metabolites stimulated by physical, chemical or pathogenic organisms such as diseases and drugs, and safety evaluation of food and drugs. Mass spectrometry (MS) coupled with gas chromatography (GC) is widely used in the complex structure analysis of biological samples [17]. Metabolic studies of fever have mainly focused on biohumoral samples and tissues, such as serum, plasma, urine, brain and hypothalamus [18,19,20,21]. Serum samples are easy to obtain, free from fibrinogen, and contain more than 1000 endogenous small molecule metabolites, which is an important metabolomic body fluid sample.

In this study, we collected serum samples from fever rats and performed untargeted metabolomics research on a GC-MS platform to explore biomarkers of yeast-induced hyperthermia in rat serum. The levels of IL-6, TNF-α, MDA and SOD in serum and the levels of PGE2 and cAMP in cerebrospinal fluid were detected using an ELISA kit. At the same time, the expression of P-NF-κB P65 and IKB-α in the hypothalamus was detected by Western blot. Afterwards, we found fifteen EA therapy related metabolites that may have participated in regulating the state of serum microenvironment, anti-inflammatory, and antipyretic pathways in serum. These results provide more evidence to better understand the underlying antipyretic mechanism of EA. In this study, we detected and analyzed the serum metabolites of fever rats treated with EA and revealed the mechanism of EA fraction in treating fever rats from a holistic perspective.

## 2. Results

### 2.1. Antipyretic Effects of Ellagic Acid

**Figure 2:**
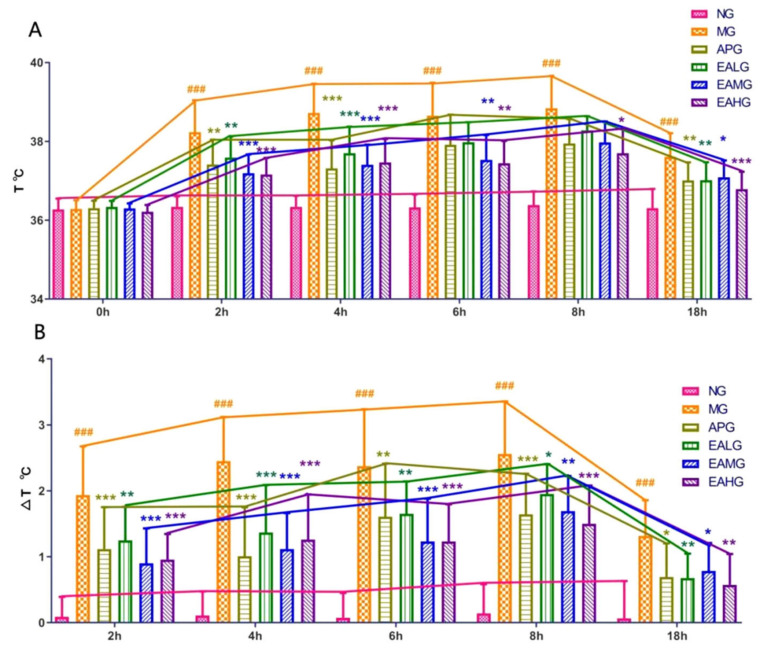
Antipyretic effects of Aspirin and EA. (**A**): The temperatures were measured at 0, 2, 4, 6, 8 and 18 h after yeast administration (*n* = 8). (**B**): Temperatures change value at 2, 4, 6, 8 and 18 h after yeast administration (*n* = 8). *p* value is for individual time point. (### *p* < 0.001, vs. NG, indicates significantly different results compared with NG; * *p* < 0.05, ** *p* < 0.01, *** *p* < 0.001, vs. MG, indicate significantly different results compared with the model that was incubated with 15 mL/kg yeast).

The temperatures of rats in each group before and after drugs administration were recorded. the temperatures were measured at 0, 2, 4, 6, 8, and 18 h after yeast administration. As shown in Figure 2, compared with NG, the temperature and the change in value of MG were significantly increased at each time point (*p* < 0.001), indicating that the yeast induced fever model was successful. After aspirin and EA administration, the temperatures and the change in value of the Aspirin group (APG), EA low dose group (EALG), EA medium dose group (EAMG) and EA high dose group (EAHG) decreased and lasted about 18 h, indicating that aspirin and EA could reduce the temperature in yeast induced fever rats. 

### 2.2. ELISA Results

Calibration curves displayed good linearity in the concentration range of 0–400 pg/mL of IL-6, the concentration range of 0–500 pg/mL of TNF-α, the concentration range of 0–2000 pg/mL of PGE2, the concentration range of 0–50 ng/mL of cAMP. The typical calibration curve equations and their correlation coefficients were calculated to be as follows:

IL-6, y = 27.34 − 115.27x + 182.22x^2^ (r = 0.9953) (1)

TNF-α, y = 1.51 + 171.88x + 182.55x^2^ (r = 0.9989) (2)

PGE2, y = (−2065.22 + 2098.96x)/(1 − 12.58x − 6.36x^2^) (3)

cAMP, y = −9.26+10.84x + 1.16/x^2^ (r = 0.9960). (4)

In the regression equation, x refers to the concentration of the analyte in serum (pg/mL) or (ng/mL), and y refers to OD of the analyte. 

**Figure 3:**
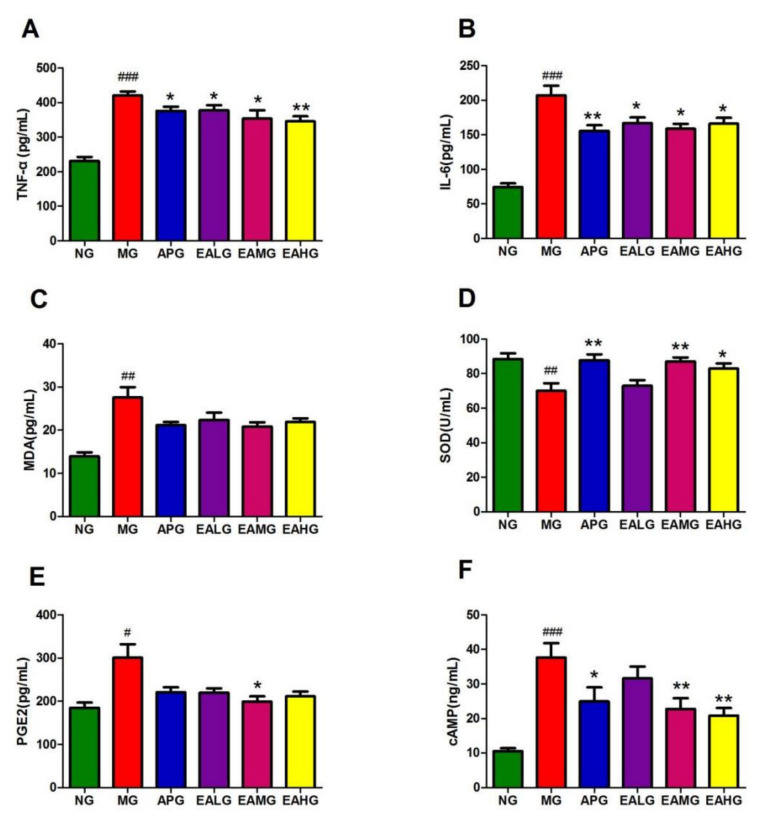
Anti-inflammation effects of aspirin and EA. (**A**): Effects of aspirin and EA in serum TNF-α secretion (*n* = 8). (**B**): Effects of aspirin and EA in serum IL-6 secretion (*n* = 8). (**C**): Effects of aspirin and EA in serum MDA secretion (*n* = 8). (**D**): Effects of aspirin and EA in serum SOD activity (*n* = 8). (**E**): Effects of aspirin and EA in cerebrospinal fluid PEG2 secretion (*n* = 8). (**F**): Effects of aspirin and EA in cerebrospinal fluid cAMP activity (*n* = 8). (# *p* < 0.05, ## *p* < 0.01, ### *p* < 0.001, vs. NG, indicates significantly different results compared with NG; * *p* < 0.05, ** *p* < 0.01, vs. MG, indicate significantly different results compared with the model that was incubated with 15 mL/kg yeast).

In serum, the concentration of inflammatory factor IL-6 and TNF-α of MG increased significantly compared to NG (*p* < 0.001) (Figure 3A,B). In the meantime, the level of IL-6 and TNF-α of APG, EALG, EAMG and EAHG decreased significantly compared to MG (*p* < 0.05), indicating that aspirin and EA could reduce the level of proinflammatory factors in the serum of yeast induced fever rats and suggesting antipyretic and anti-inflammatory effects. the concentration of MDA and SOD of MG increased significantly compared to NG (*p* < 0.01) (Figure 3C,D). In the meantime, except for EALG, the levels of SOD of APG, EAMG and EAHG decreased significantly compared to MG (*p* < 0.05). The results showed that aspirin and EA could regulate the level of oxidative stress index in the serum of yeast induced fever rats. 

The PGE2 and cAMP levels of MG in rat cerebrospinal fluid (CSF) increased compared to NG (*p* < 0.05) (Figure 3E,F). After drug administration, except for EALG, the concentration of cAMP decreased compared to MG (*p* < 0.05). 

### 2.3. Expression of P-NF-κB P65 and IKB-α in Hypothalamus

**Figure 4:**
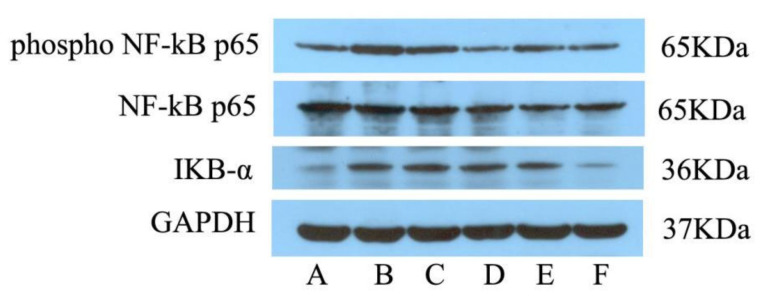
Effects of ellagic acid on P-NF-κB p65 and IKB-α protein expression in hypothalamus tissue of pyretic rats induced by brewer’s yeast as measured by Western blot. A~F:NG, MG, APG, EALG, EAMG, EAHG.

As seen in Figure 4, the administration of yeast led to a significant increase in hypothalamus P-NF-κB P65 and IKB-α levels. Treatment with EA led to a significant decrease in P-NF-κB P65 levels (*p* < 0.001), while EAMG and EAHG led to a nearly significant decrease (*p* < 0.05), suggesting that EA plays an antipyretic role by inhibiting the expression of NF-κB signaling pathway related proteins. 

### 2.4. Serum Metabolomics Profile and Multivariate Data Analysis 

#### 2.4.1. Principal Component Analysis

**Figure 5:**
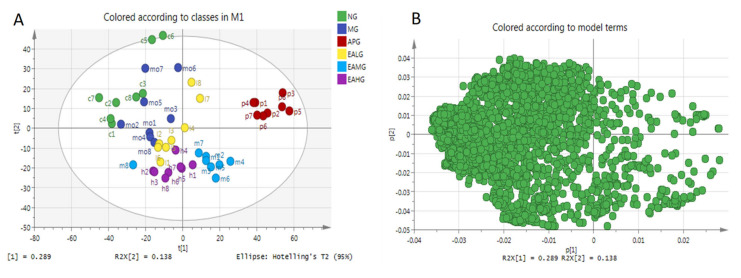
PCA of serum metabolites. (**A**): scores scatter plot. (**B**): loading.

In this study, a multivariate analysis method of Principal Component Analysis (PCA) was performed to visualize the similarities and differences among six groups. The PCA scores scatter plot was shown in Figure 5A. As we can see from Figure 5A, a good separation was demonstrated among six groups, which indicated that yeast-induced fever significantly altered the levels of endogenous metabolites in rat serum. Figure 5B is the loading of PCA. A loading diagram, also known as a correlation diagram, groups together highly correlated variables, with negatively correlated variables distributed at both ends of a straight line passing through the origin. The coordinates of each variable correspond to the correlation and directivity with PC1 and PC2 respectively. R^2^X (1) and R^2^X (2) are the explanatory rates corresponding to principal components 1 and 2. It can be seen from Figure 5B that R^2^X (1) = 0.289 and R^2^X (2) = 0.138, indicating that the contribution of the two principal components are larger. 

#### 2.4.2. Orthogonal Partial Least Squares-Discriminant Analysis

**Figure 6:**
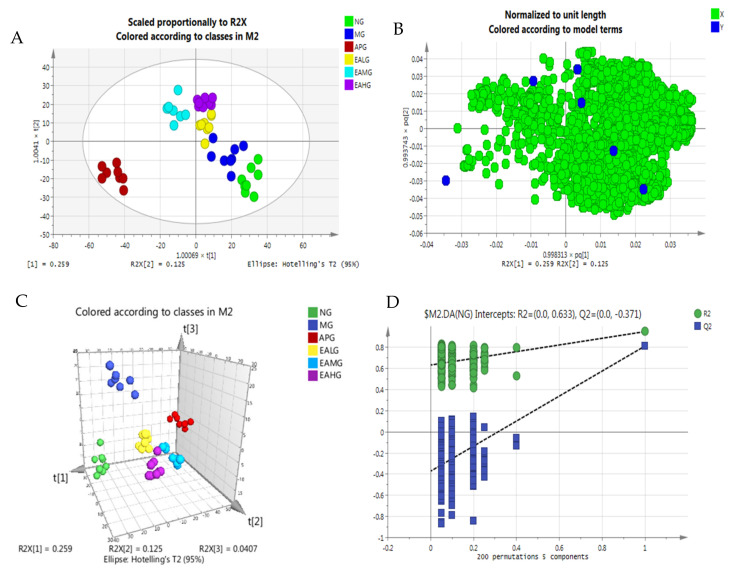
OPLS-DA of serum metabolites. (**A**): scores scatter plot. (**B**): loading of six groups. (**C**): 3D plot. (**D**): random displacement test (*n* = 200) and the explanatory rate of the model.

Orthogonal partial least-squares discriminant analysis (OPLS-DA) combines orthogonal signal correction (OSC) and the PLS-DA method to decompose the x-matrix information into y-related and y-unrelated information. Among them, the variable information related to Y is the prediction principal component, and the variable information unrelated to Y is the orthogonal principal component. Differential variables are filtered by removing irrelevant differences. The OPLS-DA scores scatter plot is shown in Figure 6. As we can see from Figure 6, a good separation was demonstrated among six groups. Under the established OPLS-DA model, the random replacement test (*n* = 200), the explanatory rate (R^2^y), prediction ability (Q^2^) and other parameters were evaluated, and the results of the permutation experiment were R^2^ = 0.633, Q^2^ = −0.371. The results show that the model has good stability and prediction ability, and the model has not been fitted. 

#### 2.4.3. Identification of Potential Biomarkers and the Changing Trends among Six Groups

**Figure 7:**
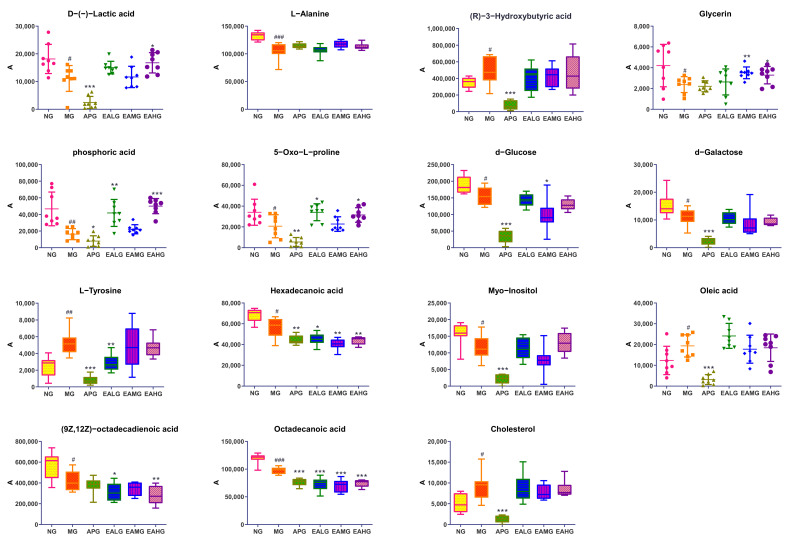
Box diagram of 15 differential metabolites in serum # *p* < 0.05, ## *p* < 0.01, ### *p* < 0.001 vs. NG; * *p* < 0.05, ** *p* < 0.01, *** *p* < 0.001 vs. MG.

Potential metabolites were screened under the OPLS-DA model, and score and load plots were made to find variables with large contribution values. At the same time, combined with VIP > 1 and *p* < 0.05 as screening criteria, the corresponding retention time and characteristic mass to charge ratio were compared with NIST library, Match score > 80%, and a total of 15 differential metabolites were screened. The detailed information about the potential biomarkers is shown in Table 1 and a box plot of differential metabolites of serum are shown in Figure 7. A total of four of fifteen metabolites were elevated after yeast injection, while eleven metabolites are reduced. Additionally, EA administration significantly regulated nine of fifteen metabolites in rat serum. It is worth mentioning that five of these nine metabolites adjusted to a normal level, and the *p*-values were statistically significant (*p* < 0.05), including D-(-)-Lactic acid, glycerin, phosphoric acid, 5-Oxo-L-proline and L-tyrosine, indicating that the five metabolites mentioned above may play key roles in the antipyretic effect.

#### 2.4.4. Metabolic Pathway Analysis of the Potential Biomarkers

**Figure 8:**
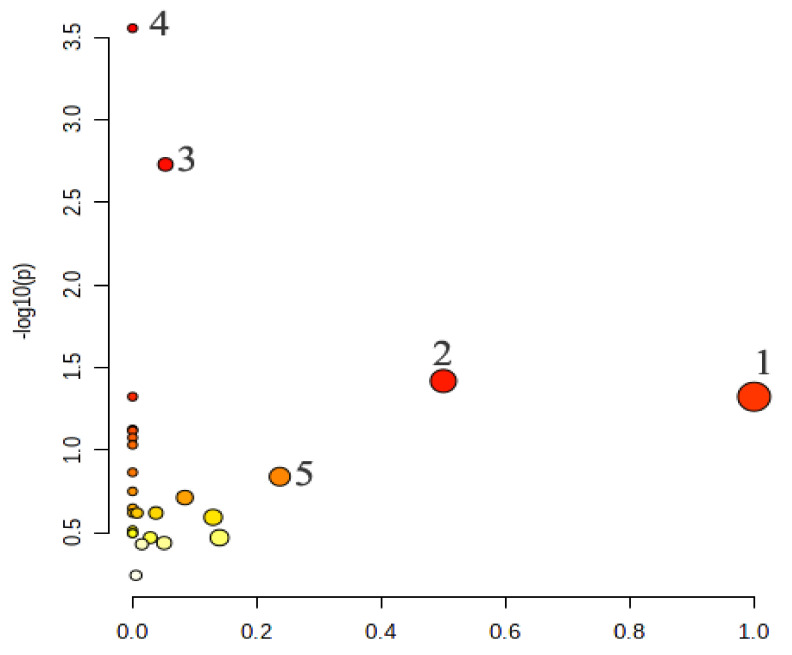
Metabolic pathway analysis. 1: Linoleic acid metabolism. 2: Phenylalanine, tyrosine and tryptophan biosynthesis. 3: Galactose metabolism. 4: Biosynthesis of unsaturated fatty acids. 5: Glycerolipid metabolism.

The metabolic pathways of 15 potential biomarkers were analyzed by the Metabo Analyst website. The results showed that the major metabolic pathways were linoleic acid metabolism, phenylalanine, tyrosine and tryptophan biosynthesis, galactose metabolism, biosynthesis of unsaturated fatty acids and glycerolipid metabolism (Figure 8). They were involved in eight potential biomarkers, including d-galactose, L-tyrosine, hexadecanoic acid, myo-inositol, oleic acid, (9Z, 12Z)-octadecadienoic acid, octadecanoic acid and glycerin. According to the analysis, EA could regulate the disorders of the four metabolic pathways, suggesting that EA has a certain degree of regulation on the metabolism disorder of the yeast-induced fever rats. 

### 2.5. Correlation Analysis between Biomarkers and Pharmacodynamic Indicators

Pearson correlation analysis was used to systematically characterize the relationship between biomarkers and pharmacodynamic indicators. Pharmacodynamic correlation analysis was conducted between fifteen different metabolites and six detection indexes of TNF-α, IL-6, MDA, SOD, PGE2 and cAMP. As shown in Table 2, there was a significant correlation between thirteen metabolites and six detection indexes. It is worth mentioning that the five components with significantly different regulatory effects of metabolites show different correlations. D-(−)-lactic acid was moderately correlated with TNF-α (*p* < 0.01); glycerin was moderately correlated with MDA (*p* < 0.01) and correlated with TNF-α (*p* < 0.01), IL-6 (*p* < 0.05), PGE2 (*p* < 0.05); phosphoric acid was moderately correlated with TNF-α (*p* < 0.01) and correlated with IL-6 (*p* < 0.05); 5-oxo-L-proline was moderately correlated with TNF-α (*p* < 0.01) and the correlation between L-tyrosine and six detection indexes was not obvious. These results indicated that the selected fever-related biomarkers had certain biological significance, and proved that it was reasonable that EA could exert an antipyretic effect by regulating them. 

## 3. Discussion

Inflammation is the body’s defensive response to stimuli and is characterized by redness, swelling, heat and pain [22]. Pro-inflammatory cytokines, such as tumor necrosis factor (TNF)-α and IL-6, are released by peripheral immune cells in response to a pathogenic challenge [23] and mediate immune responses at the site of the challenge. The NF-κB signaling pathway is involved in inflammatory regulation [24]. NF-κB transcription factor is a major regulator of inflammation and immune homeostasis. 

Nuclear factor kappa-B (NF-κB) is widely distributed in all tissues of the body and is an important transcription regulator in cells. The NF-κB family consists of five members: P65 (RelA), RelB, C-REL, P50/P105 (NF-κB1) and P52/P100 (NF-κB2). NF-κB is usually inactive in the form of p50/P65 isodimer binding to its inhibitor kappaB (IκB). When bacteria, viruses, proinflammatory factors, tumor necrosis factor, angiotensinII and other inducers stimulate body cells [25], pattern recognition receptors (PRRs), such as TLRS, NLRS, MD-2 and CD14, activate rapidly, recognize and bind the inducers, and finally form signal protein complexes and ubiquitin polymer. Activation of the IκB protein kinase complex IKK, the activated IKK phosphorylates IκB and is further ubiquitinated. The ubiquitinated IκB is released from the NF-κB dimer and degraded by the proteasome. P50/P65 is then dissociated, exposed, and nucleated to play its transcriptional activity [26]. The activation of IL-1β, IL-2, IL-6, IL-8, IL-12, I NOS, COX2, chemokines, adhesion molecules, colony-stimulating factors and so on initiate an inflammatory response. It has been shown that the NF-κB mechanisms in human PBMC are involved in staphylococcal enterotoxin A (SEA)-induced fever. SEA can induce the transfer of NF-κB from cytoplasm to nucleus in PBMC, and promote the increase of interleukin 1-β, IL-6 and tumor necrosis factor-α (TNF-α) levels in supernatant [27]. Eupafolin decreased the expression of TLR4 downstream protein and NF-κB pathway protein, and inhibited the secretion of TNF-α, IL-1β and IL-6 Eupafolin protected against cerebral I/R injury in rats, and the underlying mechanism may be associated with the suppression of apoptosis and inflammation via inhibiting the TLR4/nF-κB signaling pathway [28].

In this study, we first monitored the temperature and its changing value in pyretic rats. The concentration of inflammatory factors IL-6, TNF-α, MDA, SOD, PGE2 and cAMP increased after the injection of yeast, which were regulated after EA administration, indicating that EA plays an antipyretic role by reducing central heat mediators and inflammatory factors. WB results show that (in the hypothalamus) P-NF-κB P65 and IKB-α levels increased after successful modeling of a fever model, while the treatment with EA led to a significant decrease in P-NF-κB P65 levels, suggesting that the inducers are recognized and bound to the pattern recognition receptors on yeast cells, which activate IKB-α and are further ubiquitinated. The ubiquitinated IKB-α is released from NF-κB dimer, and p65 is exposed and nucleated to activate IL-6, TNF-α, MDA, SOD, PGE2 and cAMP inflammatory factors and activate the inflammatory response. EA decreased the level of inflammatory factors by inhibiting the protein contents of IKB-α, P-NF-κB P65 and NF-κB p65 in the NF-κB pathway. 

Fifteen different metabolites were screened in the serum of fever rats, and nine metabolites could be regulated by EA. The *P* values of five metabolites including D-(−)-lactic acid, glycerin, phosphoric acid, 5-oxo-L-proline and L-tyrosine were statistically significant. Pearson correlation analysis was used to systematically characterize the relationship between five metabolites and six detection indexes of TNF-α, IL-6, MDA, SOD, PGE2 and cAMP. The results show that four metabolites were negatively correlated with TNF-α, and *p* values were statistically significant except for L-tyrosine. In addition, glycerin was negatively correlated with IL-6, PGE2 and MDA, while phosphoric acid was negatively correlated with IL-6. Combining the results of Table 1 and Table 2, after yeast induced fever in rats, the levels of D-(−)-lactic acid, glycerin, phosphoric acid, and 5-oxo-L-proline all decreased; at this time, TNF-α, IL-6, PGE2 and MDA were in an elevated state. After treatment with EA, the levels of four metabolites increased, while the levels of TNF-α, IL-6, PGE2 and MDA decreased. These results suggest that the biomarkers selected have biological significance and it is reasonable for EA to play an antipyretic role by regulating these biomarkers. In the study of the effects of cerebroventricular administration of hyperoncotic/hyperosmotic agents on edematous brain tissue in rats with experimental head trauma, the levels of MDA and IL-1β decreased after glycerin treatment [29]. These results are consistent with our experimental results. 

The metabolic pathways of 15 potential biomarkers were involved in linoleic acid metabolism, phenylalanine, tyrosine and tryptophan biosynthesis, galactose metabolism, the biosynthesis of unsaturated fatty acids and glycerolipid metabolism. The eight potential biomarkers, including d-galactose, L-tyrosine, hexadecanoic acid, myo-inositol, oleic acid, (9Z, 12Z)-octadecadienoic acid, octadecanoic acid and glycerin hit metabolic pathway. Studies have shown that when pyrogens act on temperature regulation in the anterior hypothalamus of central neurons, the inhibitory effect stops, fat decomposition accelerates, and heat production increases [30]. Significantly elevated levels of glycerol, palmitic and oleic acids are associated with lipid breakdown, possibly caused by the role of PGE2 in preoptic area (POA) [31]. 

**Figure 9:**
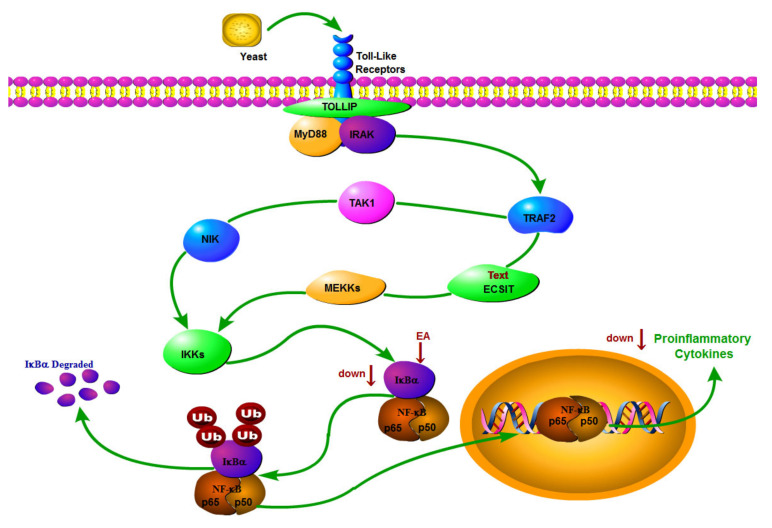
EA plays an antipyretic and anti-inflammatory role by regulating IKB-α/NF-κB pathway and inhibiting inflammatory factors.

**Figure 10:**
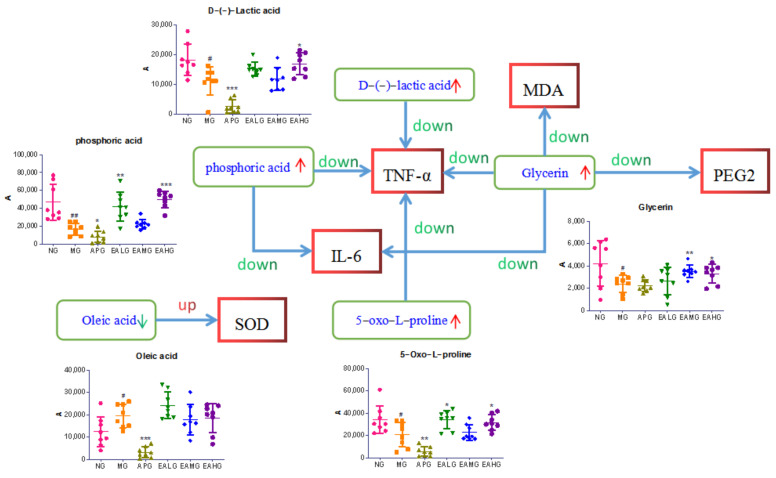
Potential target metabolic pathway of EA in the treatment of fever in rats. The green box shows the differential metabolites, ↑ level up, ↓ level down; The red box shows the regulated inflammatory factors. # *p* < 0.05, ## *p* < 0.01 vs. NG; * *p* < 0.05, ** *p* < 0.01, *** *p* < 0.001 vs. MG.

In summary, EA exerts antipyretic and anti-inflammatory effects mainly through two pathways. First, in Figure 9, EA inhibited the expression of IKB-α protein, blocked the translocation of NF-κB P65 from cytoplasm to nucleus, and reduced the production of TNF-α and IL-6 downstream proinflammatory factors, suggesting that the antipyretic and anti-inflammatory effects of EA may be realized through the inhibition of the IKB-α/NF-κB signaling pathway. Secondly, the 15 potential biomarkers mainly belong to amino acids, carbohydrate and lipids, involving five metabolites, and their mechanism of action may be related to amino acid metabolism, carbohydrate metabolism and lipids metabolism, showing target polymorphism, which is in line with the overall view of multi-component, multi-target and multi-pathway synergistic effect of modern medicine, as shown in Figure 10.

## 4. Materials and Methods

### 4.1. Reagents and Materials

Ellagic acid (EA) was purchased from Shanghai yuanye Bio-Technology Co., Ltd. (no. G26J11L119602, Shanghai, China). Yeast (*Saccharomyces cerevisiae*) was purchased from Sigma-Aldrich (no. BCBL8059V, Saint Louis, USA). IL-6, TNF-α, cAMP and PGE2 ELISA kits were purchased from Elabscience (no. 69R1TISSVG, no. 3AV258TX9Q, no. XR4SSIV8J6, no. BSIB67QHK1, Wuhan, China). MDA and SOD ELISA kits were purchased from Nanjing Jiancheng Bioengineering Institute (no. 20210601, Nanjing, China) and 4% paraformaldehyde general purpose tissue fixator was purchased from Hefei Baisha Biotechnology Co., LTD (no. 71020900, Hefei, China). GAPDH, NF-κB p65 were purchased from Servicebio (no. GB12002, no. GB12142, Wuhan, China). P-NF-κB p65 was purchased from Cell Signaling Technology (no. 13346, Boston, MA, USA) and IKB-α was purchased from BIOSS (no. bs-1287r, Beijing, China). 

### 4.2. Animals and Fever Model Processes

Male Sprague Dawley rats (150 ± 20 g) were obtained from Hunan SJA Laboratory Animal Co., Ltd (Changsha, China). All rats were acclimated for 5 days in a controlled room with temperature (23 ± 2 °C) and humidity (55 ± 10%). The rats were kept in non-toxic, high pressure, high temperature and corrosion resistant plastic cages with no more than 6 rats per cage. The rats were fed with SPF maintenance feed, and were free to eat and drink pure water. 

Forty-eight healthy male rats were selected and the temperature was measured twice by digital thermometer at an interval of 1h. A total of 48 rats were randomly divided into 6 groups: Normal group (NG, *n* = 8, 10 mL/kg 0.5% Carboxymethylcellulose sodium (CMC-Na) i.g.); Model group (MG, *n* = 8, 10 mL/kg 0.5%CMC-Na i.g.); Aspirin group (APG, *n* = 8, 100 mg/kg Aspirin i.g.), Low dose Ellagic acid group (EALG, *n* = 8, 25 mg/kg Ellagic acid suspension i.g.), Medium dose Ellagic acid group (EAMG, *n* = 8, 50 mg/kg Ellagic acid suspension i.g.) and High dose Ellagic acid group (EAHG, *n* = 8, 100 mg/kg Ellagic acid suspension i.g.), administered once a day, for consecutive 11 d. The temperature of rats was measured twice, 15 min apart, and the average of the two temperatures was taken as the initial temperature.

The rats of MG, APG, EALG, EAMG and EAHG groups were subcutaneously injected with 20% aqueous suspension of yeast (15 mL/kg) in their back. The temperatures were measured for 0 h, 2 h, 4 h, 6 h, 8 h and 18 h after yeast injection with a digital thermometer. the temperature and its change value were recorded (i.e., the difference between the temperature value and the initial temperature).

### 4.3. Samples Collection and Preparation

#### 4.3.1. Serum Samples

After the last surface temperature measurement, all rats were anesthetized by peritoneal injection of chloral hydrate. The abdominal aorta blood was immediately collected in heparinized tubes. After standing for 1 h, the tubes were centrifuged at 3000 rpm for 10 min, and supernatant was obtained, separated and stored in refrigerator at −80 °C. For measuring cytokine levels in serum, IL-6, TNF-α, MDA and SOD ELISA kits were used, following the manufacturer’s instructions. 

#### 4.3.2. Cerebrospinal Fluid

After blood collection, the rats were sacrificed by acute blood loss, and the cerebellar medullary cisterns were immediately peeled and exposed, and cerebrospinal fluid was extracted with a 1 mL syringe. PGE2 and cAMP levels in rat CSF were determined according to the kit instructions.

#### 4.3.3. Hypothalamic Tissue Samples

The hypothalamus of each rat was weighed, collected and stored at −80 °C until Western blot analysis. Sample processing was performed on ice.

### 4.4. Hypothalamic Western Blot

Hypothalamus tissue was cut into small fragments, cytoplasmic protein extraction reagents A and B were mixed at a ratio of 20:1, and phenylmethylsulfonyl fluoride (PMSF) was added to prepare 1 ml tissue homogenate. The lysis fluid was added at a rate of 200 microl per 60 mg of tissue. The maximum speed was 5 s and the ice bath was 15 min. It was centrifuged at 2000× *g* for 5 min at 4 °C for machine homogenization to obtain supernatant. Five × reduced protein loading buffer was added to the protein solution in the ratio of 4:1, and was denatured in a boiling water bath for 15 min. The protein solution of 5 μL was mixed with 10% SDS buffer and separated by 30% polyacrylamide gel electrophoresis. The protein was transferred to a polyvinylidene difluoride (PVDF) membrane and was incubated in the incubation tank of Tris Buffered Saline with Tween-20 (TBST) for 30 min. GAPDH, P-P65, P65 and IKB-α primary antibody was added at a ratio of 1:1000 and incubated at 4 °C overnight in a shaker; the second antibody was diluted with TBST in the ratio of 1:5000 and was incubated at room temperature for 30 min. The protein bands were observed using an enhanced chemiluminescence detection system.

### 4.5. The Serum Sample Processing and GC-MS Analysis

#### 4.5.1. The Serum Sample Processing

Serum samples stored in the refrigerator at −80 °C were thawed, 100 μL of serum was taken into a 2 mL EP tube, 350 μL methanol was added and centrifuged at 12,000 r/min for 10 min, then 350 μL supernatant was placed into a 2 mL EP tube and dried with nitrogen. Then, 80 μL methoxylamine hydrochloride reagent (dissolved in 20 mg/mL pyridine) was added, mixed gently, and heated in a water bath at 70 °C for 2 h. Quickly, 100 μL BSTFA (containing 1%TCMS) was added to each sample, and continued to be heated in a 70 °C water bath for 1 h, cooled to room temperature, centrifuged at 12,000 r/min for 10 min, and 100 μL was taken for testing. 

#### 4.5.2. GC-MS Analysis Conditions

GC-MS analysis was performed using the Agilent 7890B gas chromatograph system coupled with Agilent 5977B mass spectrometer. Each 1 µL of derivatized sample was injected and separated with DB-170 GC column (0.025 mm × 30 m × 0.032 µm, Agilent, San Jose, CA, USA) in split mode with a 5:1 ratio. Helium was used as the carrier gas and was maintained at a constant flow of 2 mL/min. The GC temperature method is as follows: 60 °C for 1 min; increased to 260 °C at 8 °C/min, hold for 2 min; then, increased to 280 °C at 5 °C/min, hold for 5 min.

EI ionization, collision energy 70 eV; Ion source temperature 230 °C, interface temperature 280, four-stage rod temperature 150 °C, full scanning mode, scanning range of 50–500 *m/z* and a time range of 4.5–37 min.

### 4.6. Data Processing and Statistical Analysis

The statistical significance of the identified metabolites was assessed using IBM SPSS Statistics 20. Therefore, *p* < 0.05 was set as the level of statistical significance. All statistical analysis was performed by using Prism 8 (GraphPad 8.00).

The obtained GC-MS raw data is converted into mzML format by Proteowizard. Using XCMS online processing software (http://xcmsonline.scripps.edu, accessed on 1 September 2021), peak extraction of spectra were collected, and peak identification, peak filtering operation, such as alignment, including retention time, mass-to-charge ratio and peak area of dataset, after Excel normalized processing, were imported into SIMCA14.1 (Umea, Sweden) software, and PCA and OPLS-DA multivariate statistical analyses were performed. Differential metabolites were screened according to variable importance projection (VIP) > 1. The NIST database was used for substance identification (Match > 800), and the intersection of VIP > 1 and Match > 800 were selected as differential metabolites. Metabolite pathway analysis was performed using Metaboanalyst (https://www.metaboanalyst.ca/) accessed on 23 September 2021.

## 5. Conclusions

In this study, we first investigated the antipyretic and anti-inflammation effects of EA by measuring temperature and inflammatory cytokines, respectively. Secondly, we examined the expression of P-NF-κB P65 and IKB-α proteins in the hypothalamus. After EA treatment, the levels of P-NF-κB P65 and IKB-α decreased, inhibiting inflammatory cytokines. Afterwards, we performed research on the serum metabolic profiling and biomarkers of yeast-induced fever in rats. We selected 15 metabolites as potential biomarkers of yeast-induced fever and nine metabolites as biomarkers of antipyretic mechanism of EA. The metabolic pathways of 15 potential biomarkers were involved in linoleic acid metabolism, phenylalanine, tyrosine and tryptophan biosynthesis, galactose metabolism, the biosynthesis of unsaturated fatty acids and glycerolipid metabolism. Finally, we conducted Pearson correlation analysis between 15 different metabolites and six inflammatory factors, which highlighted 13 different metabolites that were correlated. It is worth mentioning that D-(−)-lactic acid, glycerin, phosphoric acid, and 5-oxo-L-proline were negatively correlated with TNF-α; in addition, glycerin was negatively correlated with IL-6, PGE2 and MDA, and phosphoric acid was negatively correlated with IL-6.

EA inhibits the expression of IKB-α protein and reduces the production of downstream pro-inflammatory factors, suggesting that the antipyretic and anti-inflammatory effects of EA are related to the inhibition of the IKB-α/NF-κB signaling pathway. In addition, combined with the characteristics of multiple pathways and multiple targets of metabolites, the antipyretic and anti-inflammatory effects of EA are also related to amino acid metabolism, sugar metabolism and lipid metabolism.

In conclusion, this work was of significant importance in gaining insight into the serum metabolomics of pyretic rats and exploring the metabolic mechanism of the antipyretic effect of EA, which also provides a direction for the development of new antipyretic drugs.

## Figures and Tables

**Table 1:** The information and changing trend of potential biomarkers in six groups.

NO.	HMDB	t_R_/min	Metabolite	Formula	*m*/*z*	KEGG	*p*	Match Score %	NG vs. MG	MG vs. APG	MG vs. EALG	MG vs. EAMG	MG vs. EAHG
1	HMDB0001311	5.909	D-(−)-Lactic acid	C_3_H_6_O_3_	45, 29, 27	C00256	0.013	92.64	↓	↓	↑	↑	↑
2	HMDB0000161	6.457	L-Alanine	C_3_H_7_NO_2_	44, 42, 28	C00041	0.001	81.59	↓	↑	↑	↑	↑
3	HMDB0000011	7.447	(R)-3-Hydroxybutyric acid	C_4_H_8_O_3_	45, 43, 60	C01089	0.042	92.19	↑	↓	↓	↓	↓
4	HMDB0000131	8.993	Glycerin	C_3_H_8_O_3_	61, 43, 44	C00116	0.042	91.77	↓	↓	↑	↑	↑
5	HMDB0002142	10.318	phosphoric acid	H_3_O_4_P	299, 73, 314	C00009	0.003	90.64	↓	↓	↑	↑	↑
6	HMDB0000267	14.767	5-Oxo-L-proline	C_5_H_7_NO_3_	84, 28, 41	C01879	0.040	85.41	↓	↓	↑	↑	↑
7	HMDB0000122	17.901	d-Glucose	C6H13NO6	75, 71, 43	C00221	0.030	92.37	↓	↓	↓	↓	↓
8	HMDB0000143	18.162	d-Galactose	C6H13NO6	75, 71, 43	C00984	0.041	89.42	↓	↓	↓	↓	↓
9	HMDB0000158	19.198	L-Tyrosine	C_9_H_11_NO_2_	107, 77, 91	C00082	0.044	81.41	↑	↓	↓	↓	↓
10	HMDB0000220	19.809	Hexadecanoic acid	C_16_H_32_O_2_	43, 73, 60	C00249	0.015	92.26	↓	↓	↓	↓	↓
11	HMDB0000211	20.221	Myo-Inositol	C_6_H_12_O_6_	73, 60, 102	C00137	0.001	90.22	↓	↑	↓	↓	↑
12	HMDB0000207	21.760	Oleic acid	C_18_H_34_O_2_	41, 55, 43	C00712	0.043	91.83	↑	↓	↓	↓	↓
13	HMDB0000673	21.848	(9Z, 12Z)-octadecadienoic acid	C_18_H_32_O_2_	67, 81, 95	C01595	0.020	81.35	↓	↓	↑	↓	↓
14	HMDB0000827	21.948	Octadecanoic acid	C_18_H_36_O_2_	43, 73, 60	C01530	0.000	92.59	↓	↓	↓	↓	↓
15	HMDB0000067	31.801	Cholesterol	C_27_H_46_O	43, 55, 386	C00187	0.011	84.56	↑	↓	↓	↓	↓

The *p*-values is NG vs. MG. ↑: up regulated; ↓: down regulated.

**Table 2:** Pearson correlation coefficient between biomarkers and pharmacodynamic indicators.

Metabolite	The Correlation Coefficient: r					
	TNF-α	IL-6	MDA	SOD	PGE2	cAMP
D-(−)-Lactic acid	−0.430 **	−0.146	−0.177	−0.143	−0.043	−0.114
L-Alanine	−0.430 **	−0.607 **	−0.490 **	+0.035	−0.234	−0.421 **
(R)-3-Hydroxybutyric acid	−0.081	+0.122	+0.145	−0.262	+0.290 *	+0.133
Glycerin	−0.389 **	−0.348 *	−0.427 **	+0.141	−0.312 *	−0.213
phosphoric acid	−0.494 **	−0.285 *	−0.222	−0.013	−0.104	−0.269
5-Oxo-L-proline	−0.431 **	−0.181	−0.118	−0.088	+0.086	−0.129
d-Glucose	−0.241	−0.251	−0.027	−0.212	+0.046	−0.073
d-Galactose	−0.162	−0.328 *	−0.084	−0.078	−0.079	−0.145
L-Tyrosine	+0.124	+0.277	+0.216	−0.181	+0.220	+0.159
Hexadecanoic acid	−0.289 *	−0.391 **	−0.282	−0.037	+0.072	−0.197
Myo-Inositol	−0.396 **	−0.157	−0.182	−0.092	+0.102	−0.019
Oleic acid	+0.156	+0.197	+0.201	−0.321 *	+0.240	+0.189
(9Z, 12Z)-octadecadienoic acid	−0.243	−0.411 **	−0.192	+0.085	+0.004	−0.258
Octadecanoic acid	−0.331 *	−0.377 **	−0.22	−0.009	+0.006	−0.131
Cholesterol	+0.097	+0.340 *	+0.209	−0.298 *	+0.255	+0.196

“+” Positive correlation, “−” Negative correlation. ** *p* < 0.01, * *p* < 0.05. |r| ≥ 0.8, a significant correlation; |r| ≥ 0.6, high correlation; 0.4 ≤ |r| < 0.6, moderate correlation; 0.2 ≤ |r| < 0.4 low correlation; |r| < 0.2, irrelevant.

## Data Availability

The data presented in this study are available on request from the corresponding author. The data are not publicly available due to the first author is still a PhD student and has not graduated yet, relevant research is continuing.
